# “Doctor, please tell me it’s nothing serious”: an exploration of patients’ worrying and reassuring cognitions using stimulated recall interviews

**DOI:** 10.1186/1471-2296-15-73

**Published:** 2014-04-23

**Authors:** Esther Giroldi, Wemke Veldhuijzen, Alexandra Mannaerts, Trudy van der Weijden, Frits Bareman, Cees van der Vleuten

**Affiliations:** 1 Department of Family Medicine, Maastricht University, School for Public Health and Primary Care (CAPHRI), P.O. Box 616, Maastricht, The Netherlands; 2 Department of Educational Development and Research, Maastricht University, School of Health Professions Education (SHE), P.O. Box 616, Maastricht, The Netherlands; 3 Department of General Practice, Erasmus University Medical Centre, P.O. Box 2040, Rotterdam, The Netherlands

## Abstract

**Background:**

Many patients who consult their GP are worried about their health, but there is little empirical data on strategies for effective reassurance. To gain a better understanding of mechanisms for effective patient reassurance, we explored cognitions underlying patients’ worries, cognitions underlying reassurance and factors supporting patients’ reassuring cognitions.

**Methods:**

In a qualitative study, we conducted stimulated recall interviews with 21 patients of 12 different GPs shortly after their consultation. We selected consultations in which the GPs aimed to reassure worried patients and used their videotaped consultation as a stimulus for the interview. The interviews were analysed with thematic coding and by writing interpretive summaries.

**Results:**

Patients expressed four different core cognitions underlying their concerns: ‘I have a serious illness’, ‘my health problem will have adverse physical effects’, ‘my treatment will have adverse effects’ and ‘my health problem will negatively impact my life’. Patients mentioned a range of person-specific and context-specific cognitions as reasons for these core cognitions. Patients described five core reassuring cognitions: ‘I trust my doctor’s expertise’, ‘I have a trusting and supporting relationship with my doctor’, ‘I do not have a serious disease’, ‘my health problem is harmless’ and ‘my health problem will disappear.’ Factors expressed as reasons for these reassuring cognitions were GPs’ actions during the consultation as well as patients’ pre-existing cognitions about their GP, the doctor-patient relationship and previous events. Patients’ worrying cognitions were counterbalanced by specific reassuring cognitions, i.e. worrying and reassuring cognitions seemed to be interrelated.

**Conclusions:**

Patients described a wide range of worrying cognitions, some of which were not expressed during the consultation. Gaining a thorough understanding of the specific cognitions and tailoring reassuring strategies to them should be an effective way of achieving reassurance. The identified reassuring cognitions can guide doctors in applying these strategies in their daily practice.

## Background

Many patients consult their general practitioner (GP) because they experience certain symptoms and are worried that these may be indicative of serious illness [1-3]. Patients with a chronic or progressive condition often worry about the potential impact of disease on their daily life, such as becoming dependent on others [4]. Anxiety about illness causes psychological distress, posing a major burden to patients [5,6]. Understanding how to reassure patients effectively is important because reassurance can improve patients’ health status and well-being [7-11]. It has also been suggested that effective reduction of anxiety helps patients to better understand information given by the doctor and strengthens the doctor-patient relationship [8,10].

As general practitioners are generally aware of the importance of effective reassurance, they use various strategies to reassure their patients, such as enhancing patients’ insight into the benign cause of the complaint ([12], Giroldi E, Veldhuijzen W, Leijten C, Welter D, Muris J, van der Weijden T, van der Vleuten CPM: ‘No need to worry’: exploring family physicians’ expertise in reassuring patients: Submitted.). However, evidence for the effectiveness of these strategies is limited. Previous studies have shown that attempts to reassure patients are often ineffective [13-16]. Clinicians and textbooks may attribute unsuccessful reassurance to patients’ abnormal illness behaviour, i.e. persistence of illness behaviour despite medical reassurance [13,17-19]. However, an alternative explanation placing less blame on the patient is that ineffective reassurance may be due to the fact that patients and doctors perceive clinical encounters in different ways [20-22]. Patients might understand and interpret the doctor’s reassuring statements quite differently than expected by the doctor. For example, the doctor stating that the complaints are not serious fails to achieve the intended effect when these complaints severely impact the patient’s everyday life [17]. This suggests that a thorough understanding of patients’ concerns is a key factor for successful reassurance. Patients do indeed feel more reassured when their doctor explores their concerns using screening questions such as ‘have you any other concerns or questions?’ [10].

The “common sense model of illness representations” describes how patients facing a health problem construct disease cognitions related to symptoms, causes, duration, controllability and consequences [23-26]. The emotional component of illness comprises how patients feel in response to these cognitions, e.g. their being anxious or worried [24]. Effective reassurance is likely to depend on doctors being aware of and addressing these cognitions and worries. This supports the relevance of studies aimed at enhancing insight into mechanisms that determine effective reassurance of patients. Therefore we firstly aim to understand what cognitions underlie patients’ worries, what cognitions underlie patients’ feeling of being reassured and what factors (GP factors, communication factors and other contextual factors) support patients’ reassuring cognitions.

## Methods

### Design

In order to explore patients’ experiences and cognitions related to concerns and reassurance, we conducted a qualitative study in which we used recent, videotaped consultations of the participating patients with their GP as stimulus for stimulated recall interviews with the patients [27]. A thematic analysis of the interviews was performed to identify common themes. Drawing upon principles of grounded theory, we conducted an iterative process of data collection and analysis to facilitate further exploration of themes in subsequent interviews and the method of constant comparison [28].

### Ethical approval and informed consent

The Medical Ethical Commission of Maastricht University Medical Centre granted ethical approval for the study protocol. All participating patients gave informed consent. Codes were used to anonymize the verbatim transcriptions of the recorded interviews.

### Study context

The study was conducted in Dutch general practices. The Netherlands has general practices with enlisted patients, with each patient allowed to register in and attend one practice only. Thus as a general rule, GPs have a continuous and longstanding relation with their patients. In the visited practices, patients’ appointments are booked in advance.

### Selection procedures

GPs were recruited and informed of a study on reassurance with an invitation letter and a follow-up telephone call. Twelve GPs agreed to participate and were visited during a morning clinic. A total of 134 patients were informed of a study on doctor-patient communication. One of the researchers informed patients in the waiting room, both verbally and with an information letter. Before and after their consultation with their GP, the 68 participating patients (50.7%) rated the level of their concern (i.e. how worried about your health problem are you at this moment?) on a scale ranging from 0 (not worried at all) to 10 (very worried). After the consultation, the GPs rated for each patient how important achieving the goal of reassurance was in this particular consultation, also on a scale ranging from 0 to 10. Consultations were observed and recorded on video to enable patients to reflect upon non-verbal communication. From this sample we aimed to select two consultations at each visit that combined a high rating by the GP on the importance of reassurance with a high rating by the patient on their level of concern before the consultation. In case we were unable to identify two of such consultations, we prioritized the GP rating. Reassurance needed to be an explicit goal of the GP since the selected consultations were to be used for interviews with GPs about the way they reassured patients and with patients about how they experienced their GP’s reassuring efforts.

### The interviews

The selected patients were interviewed by trained interviewers, preferably on the day of the consultation or shortly thereafter. To guarantee that patients felt comfortable, they were interviewed in their own homes. To ensure consistency in the approach of the three interviewers, they thoroughly discussed the interview procedure before and during the period of data collection. Moreover, a rehearsal session with simulated patients was organised to practice, discuss and receive feedback on the stimulated recall procedure.

At the start of the interview the patients were invited to elaborate on their health concerns in relation to their GP visit. Next, the researcher and the patient watched the recording of the patient’s consultation. The patients were invited to stop the tape whenever they felt that something occurred that had influenced their level of concern during the consultation and to recall their thoughts regarding this moment. The researcher could stop the tape too at moments she considered relevant and ask patients how they experienced these moments. The researchers made this decision based on their literature study and clinical experience and by paying close attention to (non)-verbal expressions of cues and concerns. The researcher prompted patients to elaborate on their experiences and thoughts (e.g. why was that reassuring; what were your thoughts at that moment?).

### Data analysis

The interviews were transcribed verbatim. After carefully reading the transcripts, the researchers used specialized software (Atlas-ti) to identify and code relevant fragments. Open coding was used to identify themes and axial coding was performed to understand how themes related to each other. Transcripts were compared with those that had already been analysed to revise and refine themes. Memo writing guided the identification and interpretation of patterns that emerged from the data [28]. The coded sections and memo’s formed the basis for writing interpretive summaries of every patient. These summaries helped to understand how different worrying cognitions were counterbalanced by reassuring cognitions and which factors facilitated that process. To increase the credibility of the results, the transcripts were coded independently by at least two researchers with different backgrounds, i.e. health sciences (EG) or medicine (WV/AM), increasing scope and deepening understanding of the data [29]. Discrepancies in coding were discussed until consensus was reached. All the authors discussed the themes that were identified during the analysis.

## Results

### Data characteristics

A total of 21 patients were interviewed (Table 1), of which nine were female. Patients’ age ranged from 19 to 89 years (mean: 53.8 years). The twelve GPs came from ten different practices, representing a range of practice settings (rural, urban, solo, duo and group practices). The sample represented a mixture of follow-up and stand-alone appointments, with a mean consultation time of 12 minutes (Table 1). The analysis of the GP interviews indicated that GPs did not expect serious pathology to be present in any of the selected consultations, based upon their findings of the history taking, physical examination and, if applicable, additional investigations.

**Table 1:** Patient and consultation characteristics

**Patient**	**Patients’ rating: concern pre-consultation (0–10)**	**GPs’ rating: importance reassurance (0–10)**	**Complaint(s)**	**Stand-alone/follow-up appointment**	**Duration consultation(minutes)**
1	10	8	Chest pain	Stand-alone	16
2	6	8	Abdominal pain, lower back pain	Follow-up	9
3	0	8	Reduced kidney function	Stand alone	9
4	6	10	Hypertension, lower back pain	Follow-up	15
5	3	10	Stool problems	Follow-up	8
6	10	10	Accelerated heartbeat, headache	Follow-up	19
7	10	10	Chest pain, headache, hypertension	Stand-alone	15
8	0	3	Burn	Stand-alone	7
9	1	4	Soar throat	Stand-alone	7
10	4	7	Sudden shaking attack, furuncle	Follow-up	7
11	7	7	Hypertension, headache, nausea	Stand-alone	14
12	2	8	Chest pain	Stand-alone	13
13	8	7	Skin mark	Follow-up	10
14	4	7	Ankle pain	Stand-alone	9
15	10	10	Breast lump, joint pain	Stand-alone	17
16	6	8	Burn out, hypertension	Follow-up	21
17	1	7	Wrist pain, excessive sweating	Stand-alone	8
18	7	10	Hypertension, weight gain	Follow-up	12
19	7	9	Hip complaints	Stand-alone	11
20	1	9	Urinary problems	Stand-alone	11
21	0	9	Leg pain	Stand-alone	15

As the analysis of the last five interviews did not yield any new themes and confirmed the results, saturation was reached.

### The interviews

Twenty patients were interviewed on the day of the consultation and one patient was interviewed seven days later. The interviews lasted between thirty and sixty minutes. In most cases patients spontaneously shared cognitions related to their concerns. Even patients with a low level of concern spontaneously expressed concerns and underlying worrying cognitions during the interview. Not all worrying cognitions discussed in the interview had been mentioned in the consultation. Specifically cognitions regarding the adverse effects of medication were expressed in several interviews but were never mentioned during consultations. Patients easily described the GPs’ actions they experienced as reassuring, but did not often express spontaneously how these actions supported reassuring cognitions. This was explored by further probing by the researcher.

### Worrying cognitions and how reassuring cognitions counterbalance them

Patients described four worrying core cognitions. As explanations for why they had these core cognitions, patients expressed person-specific cognitions (concerns related to their complaints, their body and self-image) and context-specific cognitions (concerns related to their social environment) (Table 2). While person-specific cognitions were often expressed in relation to context-specific cognitions, they were sometimes expressed individually.

**Table 2:** Worrying core cognitions and underlying cognitions

**Worrying core cognitions**	**Underlying cognitions**	
	**Person-specific cognitions**	**Context-specific cognitions**
**I have a serious disease**	- I have alarming symptoms/abnormal test results which indicate serious disease.	- This is a common disease according to the media/in my family/among my friends and acquaintances.
	- The symptoms I have are not normal.	- The media/my friends say that my symptoms are indicative of a serious disease.
	- Now that I am getting older the chance of having a serious disease is increasing.	- The symptoms I have must be abnormal as I know of no people in my social environment and with a similar background to mine that have such symptoms.
	- My symptoms have not disappeared after the treatment so something must be wrong.	
**My health problem will have adverse physical effects**	- I have a health problem that will lead to serious illness causing disability/additional conditions/death.	- These adverse effects happen all the time according to the media/in my family/among people I know.
	- My symptoms are getting worse.	
	- I do not know how the symptoms can be treated since I do not know what causes them.	
**My treatment will have adverse effects**	- The treatment I received was incorrect, so my health problem will persist.	- In my social environment I have seen many cases of incorrect treatment of this problem with bad outcomes.
	- If I take medication, I will have to continue to take it indefinitely/there will be side effects/I will have difficulty sticking to my drug regimen.	- In my social environment I have often seen adverse effects of medication.
**My health problem will negatively impact my life**	- Having health problems does not fit with how I see myself and my future.	- My social environment is not supportive when I have problems and concerns/does not allow me to deal with the problem in my own way.
	- I have so many health problems at the same time/My health problems are getting worse/my treatment is no longer effective.	
	- My health problems will make me dependent on others/limit my daily functioning.	

In addition, patients mentioned five reassuring core cognitions. As explanations for why they had these core cognitions, patients mentioned consultation-specific factors (GPs’ actions during the consultation) and context-specific factors (patients’ pre-existing cognitions based on experiences that had occurred before the consultation, e.g. regarding their GP, the doctor-patient relationship and prior events) (Table 3).

**Table 3:** Reassuring core cognitions and supporting factors

**Reassuring core cognitions**	**Factors supporting reassuring core cognitions**	
	**Consultation-specific factors. My doctor:**	**Context-specific factors. There is:**
**I feel safe and supported because I have a trusting relationship with my doctor.**	**- emphasizes the equality of our relationship by:** approaching me in a friendly manner, using humour.	**- an existing trusting doctor-patient relationship based on:** the duration of the relationship; I know I can always ask the doctor any question I have; the doctor being always open and honest.
	**- shows that he/she is interested in me as a person by:** allowing me to tell my story, listening attentively, exploring and understanding my feelings and personal situation.	
	**- shows involvement by:** showing empathy; monitoring my condition	
**I trust my doctor’s judgment since I trust his/her expertise.**	**- obtains a good understanding of my problems and symptoms by:** allowing me to tell my story; listening attentively; exploring and summarizing my symptoms/feelings/context; showing/telling me what he/she is typing; referring to the previous consultation.	**- an experienced doctor who:** has had many years of experience; sees many patients with problems that are similar to mine; has a good reputation in my neighbourhood.
	**- takes the necessary actions to adequately investigate my symptoms by:** exploring alarming symptoms; performing a physical examination and other investigations; referring me to a specialist.	**- a familiar doctor who:** knows my whole history.
	**- explains the reasons for his/her actions by:** announcing and explaining what he/she will do during the physical examination and which further investigations will be done.	**- a careful doctor who:** has never made a mistake; has always been quick and effective; has always referred me when it was necessary.
	**- takes care to perform actions properly and thoroughly by:** making sure that he/she does not forget anything by sharing findings with me during the examination/when typing during the consultation; discussing test results with me; proposing a good specialist.	
**I do not have a serious disease**	- **underpins why my symptoms are not serious by**: explaining (using visual tools) why my symptoms/findings are not consistent with a serious diagnosis; demonstrating how the examination excludes a serious diagnosis; emphasizing that the maximum amount of information has been obtained; pointing out that a specialist will make the same diagnosis.	**- sufficient evidence provided to exclude a serious diagnosis because:** multiple examinations, investigations and medical professionals have excluded serious disease.
	**- enables me to reassure myself by:** exploring alarming symptoms that are not present; ordering tests and investigations of which I can interpret the results.	
	**- underlines (non-)verbally that my symptoms are not serious by:** a calm and unconcerned expression; stating that I do not have a serious illness/there is no need to worry/my symptoms are benign.	
	**- shows that actions that might indicate a serious diagnosis are not really necessary by:** stating there is no need for illness behaviour; not referring urgently; emphasizing that the purpose of the referral is to re-assure me.	
	- **provides insight into my tendency to worry about physical symptoms by:** giving a cause for this tendency that I can recognize and understand, emphasizing the importance of being aware of this tendency.	
**My health problem is harmless/easy to treat.**	**- gives a logical and understandable explanation for my symptoms by:** explaining what causes them; giving an explanation that is consistent with my self-image; linking new symptoms to a benign diagnosis on which we had agreed.	
	**- clarifies the cause of my symptoms by:** using clear language; talking slowly; checking that I have fully understood the benign diagnosis; using visual tools; sharing findings during the physical examination.	
**My health problem will: disappear/not return/not deteriorate**	**- ensures I receive adequate treatment by:** tailoring the treatment to my wishes and expectations; proposing treatment that I can execute myself; treating the cause of my symptoms; referring me to (a team of) specialists for treatment	**- evidence that I am getting better because:** the symptoms are diminishing, the treatment is effective.
	**- adequately monitors my condition by:** indicating when he/she wants to schedule a follow-up visit/check-up; monitoring my condition him/herself.	**- a doctor who always carefully monitors my condition by:** scheduling regular follow-up visits; being available in case of problems between appointments.
	**- gives a positive vision of my future by:** outlining the natural course of the disease; emphasizing that everything will be done to ensure a full recovery.	

**Figure 1:**
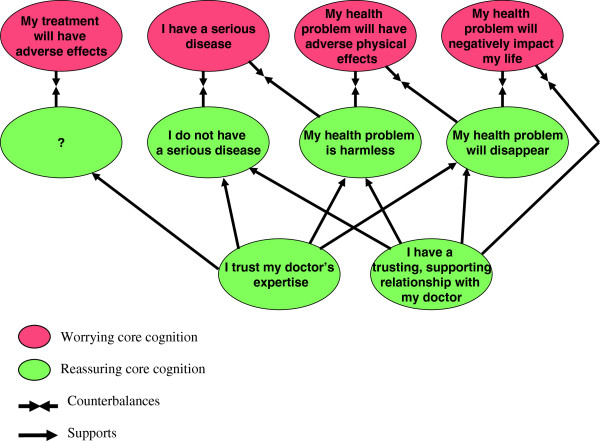
Reassuring cognitions to counterbalance worrying cognitions.

The analysis of the data revealed possible relationships between worrying and reassuring core cognitions, as three reassuring cognitions seemed to directly counterbalance either one or two specific worrying cognitions (Figure 1). However the two reassuring cognitions ‘I trust my doctors’ expertise’ and ‘I have a trusting and supporting relationship with my doctor’ appeared to mainly have an indirect reassuring effect in terms of supporting the other three reassuring cognitions. Patients mentioned these trust-related factors to be reassuring because they created an environment in which the patient felt safe and supported and accepted what the GP was saying, however they did not seem to directly counterbalance patients’ specific worrying cognitions. One exception was that the reassuring cognition ‘I have a trusting and supporting relationship with my doctor’ seemed to directly counterbalance the worrying cognition ‘My health problem will negatively impact my life. Patients mentioned no reassuring cognitions that directly counterbalanced the worrying core cognition ‘my treatment will have adverse effects’.

Following the structure of Table 2 and using illustrative quotations, we describe in some detail patients’ worrying cognitions. For every worrying cognition, we describe how these seemed to be counterbalanced by patients’ reassuring cognitions, using some specific examples from Table 3. For a complete list of cognitions see Tables 2 and 3.

### Worrying core cognition: I have a serious disease

Most of the participating patients expressed concerns that they might have a specific serious disease, such as cancer or a heart condition, often triggered by an alarming symptom, such as chest pain. Some patients said they felt that having a symptom in itself is abnormal and therefore indicative of serious disease, without having a specific disease in mind. These person-specific cognitions were often mentioned alongside context-specific cognitions, such as worrying they might have a specific severe illness because it runs in the family.

This pain persisted here and then I thought: ‘Couldn’t it be my heart?’ I wanted to know what it is actually, as there are a few in my family who died of heart disease.

Patient 12

I had a blood test showing reduced kidney function, and because my mother died from renal disease I wanted to ask the doctor what might be the matter and if this could cause problems. I was worried that it might be hereditary.

Patient 3

Patients often mentioned that they trusted their doctor’s judgement. This trust was partly based on pre-existing cognitions, i.e. cognitions arisen from previous encounters with the GP, for example about the presence of a long-lasting doctor-patient relationship.

I trust him, he has never been wrong in treating me, my husband, and my whole family. I have known him for years.

Patient 15

Often such pre-existing cognitions appeared to be triggered by what happened in the current consultation.

Here we are making jokes. You can see that we can get along very well. Therefore I trust him. I value that he knows who I am and that he is attuned to me.

Patient 10

Patients seemed to trust their doctor’s expertise when they felt the doctor had obtained a good understanding of their problem, e.g. by listening attentively to their story. Patients mentioned that the doctor needed to understand what they were worried about to be able to conduct appropriate examinations. Patients expressed that taking the necessary steps to thoroughly investigate their complaint, e.g. by performing a proper physical examination, enabled the doctor to come to an accurate diagnosis.

I was able to properly talk with him and I gave him various signs, that I was thinking ‘maybe it is the thyroid gland?’ Well, he reassured me about that. He paid attention to that and he made the effort to have a look. I thought there might be a cause, and when he said that is not the case, well, I was satisfied with that.

Patient 9

Well, it seems to me that it [physical examination] is a part of the consultation, for without it the doctor cannot find out what is going on. She also explained things to me, but I think that without a proper physical examination it is impossible to determine what the matter is.

Patient 17

Patients also expressed that they needed to be convinced that their complaint was not serious by evidence or signs that denied the presence of severe illness. Some patients said they were already able to reassure themselves by being able to answer negatively to the doctor’s questions about alarming symptoms.

As I do not have it [sweating, nausea], because I could answer negatively, I found that reassuring.

Patient 1

In addition, patients stressed the importance of the doctor explaining how test results or findings of the history taking and physical examination excluded a harmful diagnosis. Different types of explanations were mentioned. Some patients preferred such an explanation to take place during the physical examination.

In that way [describing findings during the physical examination] he won’t forget anything and I know immediately how things stand. That is quite a relief.

Patient 21

In case of an abnormal test result, patients felt reassured when the doctor used visual tools, such as anatomical models and graphs, to explain why they are reported and that they are no reason for concern.

For some patients it was sufficient to understand why the complaint was not serious, but others said they also needed an alternative explanation showing that their symptoms were harmless before they felt completely reassured. Patients believed the doctor’s explanation that their symptoms were harmless when the doctor gave a clear, logical explanation that the patient could recognize. The doctor could do so, for example, by demonstrating and explaining during physical examination that chest pains can be caused by muscle tension. Several patients mentioned that they did not need any additional investigations after receiving a clear, detailed explanation of their complaints.

When she says it [an X-ray] is not necessary and everything is fine, I trust her and I feel sufficiently reassured. So explaining this and that it is unlikely to be that and it is probably this. And that she explained really well why it was that and not something else.

Patient 14

When he explains it you start to think, obviously when I have pain in my left foot end my right hip, walking is difficult and I distribute it. Then I think, well what he says makes sense, it is quite a logical explanation.

Patient 21

In contrast, a few patients mentioned that in order to feel reassured, they needed additional tests or a referral to a specialist, after which they would obtain absolute certainty. They felt that the GP is limited in his/her diagnostic means and that additional investigations would give a more precise result than a physical examination. Some patients preferred an investigation of which they are able to understand the results.

With an X-ray I can see it myself, it’s tangible. Then I feel more reassured than with a blood test. You see I’m no expert, such a result of a blood tests is like Chinese to me.

Patient 4

Despite these investigations or referrals, patients seemed to be aware that the doctor him/herself was not worried. The doctor demonstrated this by not referring the patient urgently or by emphasizing that the only purpose of the referral is to reassure the patient.

He knows that when I don’t do it [X-ray] that I keep feeling it, keep worrying and get really nervous. He knows that and therefore he says: ‘ to reassure you I’ll order an X-ray’. Once that is done it will all be ok.

Patient 15

### Worrying core cognition: My health problem will have adverse physical effects

Worries about having a serious disease were often accompanied by worries about potential adverse physical consequences of such a disease, such as additional conditions and death.

But well, I am worried about the pressure at the back of my head. Then I think of my heart, for that is the pump and the pump is the main thing, and, to be honest, then I worry that I will not live to an old age. Yes, your heart is in fact the thing of your body and if it is not alright, other things will be wrong as well. For you get a domino effect.

Patient 4

Worries about the adverse effects of a complaint were often due to the experiences of family members and friends or to information on the internet.

I have high blood pressure with symptoms and then you hear all sorts of stories and you know people, some I know personally, who have suffered attacks due to high blood pressure and were partially paralyzed. I have two young children and I do not want that.

Patient 18

Patients who worried that their symptoms might lead to adverse consequences mentioned to feel reassured after the cause of their symptoms was explained to them. In some cases a clear explanation helped patients to understand that the symptoms were easy to treat. Having control over their complaint by being able to treat it themselves was reassuring as well.

I know what causes it. Things are in my own hand now, I know what I can do about it, and that is reassuring.

Patient 18

Patients felt reassured also when they were convinced that their complaint would disappear or when they believed it would not get worse or return. This depended on adequate treatment as well as close monitoring, preferably by their GP, who was considered to be the most capable of judging how the complaint would develop.

### Worrying core cognition: My treatment will have adverse effects

Some patients believed that treatment would lead to serious consequences. This could relate to treatment that already had been performed, in this case a hip replacement.

Your can read a lot about new hips, metal on metal can cause all sorts of things. My sister still has these complaints, pain in her leg, swollen. I have the same. She already has it [new hip] for 5 years now. Then I rather go back on time.

Patient 19

This cognition however mostly arose when the doctor prescribed medication during the consultation or when the patient thought the doctor might prescribe medication in the future.

I know someone who also has something like that and when he reads the side effects of the medication, then you start to think, do I really have to take that? Then I’m afraid that there will be side effects and they are going to make it worse.

Patient 7

Patients who worried about potential adverse effects of medication use mentioned no reassuring cognitions that counteracted these particular concerns. A few patients with these concerns did derive some reassurance from trust in their doctor’s expertise, however this did not seem to influence their specific worrying cognition. The patient worrying about a possible incorrect hip replacement did also not express any reassuring cognitions apart from trust in the doctor’s expertise, yet for a different reason. This patient felt partly reassured because her doctor referred her for an additional investigation. Inevitably the results and treatment options had not been discussed yet so the patient’s worries remained for the most part.

### Worrying core cognition: My health problem will negatively impact my life

Several patients expressed difficulties in coping with their health problems as they worried about the impact of these problems on their life, e.g. on their everyday functioning. Some of these patients had been experiencing several health problems for a longer period and some mentioned that their problems were getting worse and/or that their treatment was no longer effective. A few patients also indicated non-supportive family members, friends and working environment as one of the reasons for their experienced difficulties. Another cognition that patients expressed in relation to this type of concern was the mismatch between living with complaints and the patient’s vision of his/her future, for instance when the patient had expected to grow old without experiencing any physical problems.

Well, I find it difficult to deal with that this is the case for several things. It really makes me feel like, hey guys is there anything that is not wrong with me, just put me out with the garbage. I find this very discouraging, like, well you are getting old, shortly you will be written off completely (…). I am 65 years old and I would like to live to 85 without any problems.

Patient 2

While a trusting relationship seemed to have an indirect reassuring function for most patients, patients who struggled with the impact of their complaints on their life, explicitly described the reassurance they obtained from a trusting and supporting relationship. The doctor showing involvement and empathy and listening attentively to the patient’s story supported such a relationship. This made patients feel their doctor was interested in them as a person and was genuinely trying to help them. Especially patients who felt that people in their environment did not take them seriously appreciated naming concerns.

It is reassuring to be listened to. If I had talked about this with my boss, he would have listened to me for two seconds and told me to go to bed and see how things were in the morning. And he says, no, you should tell your story properly and I will not interrupt you. One is not afraid to have a proper talk with this man, because you know that everything you say will be heard, that he really listens to you. He gives you time to get it off your chest. He asks some pertinent questions and otherwise he keeps quiet. Well, that is why I am here isn’t it?

Patient 16

Several patients mentioned that they felt reassured when they believed their complaints would be resolved and not return. These concerns seemed to be dealt with particularly effectively, when the doctor communicated a treatment plan and a positive vision of a future without complaints.

It is very much tailored to the person. He says, well you need a little more of this. That is reassuring. Then they start to work on things you might do better in order to prevent this from happening again.

Patient 16

He had this idea, that I can go to that hospital with a team of specialists and try it [treatment] again. And I’ll try it. I’m optimistic about it, maybe they can do something positive.

Patient 21

## Discussion

### Main findings

During the interviews, patients mentioned a range of worrying core cognitions underlying their concerns: ‘I have a serious illness’, ‘my health problem will have adverse physical effects’, ‘my treatment will have adverse effects’ and ‘I find it difficult to live with my complaints’. Patients related these worrying core cognitions to a range of underlying person-specific and context-specific cognitions. Patients also described different reassuring cognitions: ‘I trust my doctor’s expertise, ‘I have a trusting and supporting relationship with my doctor’, ‘I do not have a serious disease’, ‘my health problem is harmless’ and ‘my health problem will disappear’. As reasons for these cognitions, patients mentioned the doctor’s actions during the consultation and pre-existing cognitions related to their doctor and previous events. Worrying and reassuring core cognitions appeared to be related, as worrying cognitions were counteracted by specific reassuring cognitions.

### Main findings in relation to the literature

Patients who were concerned about having a serious disease expressed to be reassured when the GP explained why the complaint was not serious and moreover gave insight into the harmless cause of the complaint. These findings are similar to our previous study in which we interviewed GPs about the strategies they used to reassure patients (Giroldi E, Veldhuijzen W, Leijten C, Welter D, Muris J, van der Weijden T, van der Vleuten CPM: ‘No need to worry’: exploring family physicians’ expertise in reassuring patients: Submitted). Both studies provide empirical support for Starcevick’s view that this is an effective strategy to reassure patients [30]. The reassuring cognitions we identified in the present study are in line with the process in which patients with medically unexplained symptoms (MUS) are supported to re-attribute somatic complaints [31,32] and with giving hypochondriac patients insight into the causes of their tendency to worry as an effective component of Cognitive Behavioural Therapy [33].

Patients also worried about potential serious consequences of complaints, such as disability. The main reassuring cognition for these patients was the belief to being able to influence their health problem. This finding provides empirical support for Buchsbaum’s description of the importance of providing patients with information, thereby enabling them to re-establish control and self-confidence [4], and for the results of a qualitative study by Andén et al. showing that ‘understanding what I have’ was the key consultation outcome for patients and that it was not sufficient to merely cure the complaint [34].

Buchsbaum also described how an empathic doctor showing genuine interest and compassion can provide support during emotionally turbulent times [4]. A compassionate doctor and exploration of patients’ concerns have been shown to reduce anxiety in cancer survivors [8] and cancer patients, respectively [10]. In our study, patients worrying about the impact of health problems on their everyday life explained how a trusting doctor-patient relationship was reassuring for them. With simple actions, the doctor provided emotional support to patients who did not receive this from their social environment [35]. Here, a trusting relationship served as the instrument of reassurance while also having an indirect supportive effect on patients with other worrying cognitions. A trusting relationship created an environment in which patients felt comfortable and were more likely to accept the doctor’s information and conclusions.

In addition to patient-centred communication skills that have received considerable attention in the literature, such as showing empathy, this study demonstrates the importance of trusting the doctor’s expertise in the acceptance of a reassuring diagnosis. Confirming the findings of our previous study, patients experienced it as reassuring when the doctor showed he or she adequately understood the complaint and took what patients believed to be the necessary action to investigate the complaint (Giroldi E, Veldhuijzen W, Leijten C, Welter D, Muris J, van der Weijden T, van der Vleuten CPM: ‘No need to worry’: exploring family physicians’ expertise in reassuring patients: Submitted). Besides the reassuring effect of the GPs’ actions in the consultation, the present study also highlighted the importance of patients’ pre-existing cognitions about their doctors’ expertise.

We described earlier in the background that patients and doctors perceived consultations differently [20-22], and hypothesized that this might lead to ineffective reassurance [13-16]. A comparison of the results of the present study with those of our earlier study nevertheless showed considerable overlap between strategies GPs believe to be reassuring and patients’ reassuring cognitions. However it cannot be concluded that this overlap will be present on the level of individual consultations. Furthermore, reassurance may be difficult to achieve in specific patient groups with high anxiety [14,16] and more feasible in the less anxious population we studied. Our findings are consistent with previous research showing that in most patients reassurance is a multifaceted process involving more than just providing normal results of diagnostic tests [13,15,36]. Most patients wanted clear explanations to help them understand their complaint, after which they felt no need for any additional diagnostic tests. A few patients, however, did not feel sufficiently reassured by their GP’s explanations and needed more investigations or a referral to a specialist. These differences between patients point to the important observation that worrying and reassuring cognitions can vary considerably among patients, requiring doctors to use situation-specific reassurance strategies. This may be challenging considering that training in medical communication skills tends to be of a quite generic nature [37]. It might be particularly challenging to address patients’ undisclosed or ambiguously expressed concerns, a phenomenon present in this study as well as in previous research [38].

Although we did not aim to explore which sorts of cognitions were or were not expressed during the consultations, we noticed that worries about the effects of medication use were never expressed. Obviously, an in-depth interview gives patient much more opportunity to elaborate on their concerns than a 10-minute consultation. Nevertheless, the reasons for patients’ unexpressed concerns in this study seemed to be related to the subject. Patients may feel uncomfortable disclosing concerns about the physician’s treatment plan.

### Strength and limitations

Interestingly, none of the patients, including those who remained worried, expressed worrying cognitions about their own doctor, e.g. regarding the absence of trust. Although we explicitly asked all patients if they could think of anything that would have reassured them more, only one patient described this. The absence of this action was however no cause of increased concern. Patients who mentioned doubts about other GPs and specialists generally remarked that their own doctor did much better. There are several possible explanations for this phenomenon. Firstly, patients may have given socially desirable answers and be reluctant or not used to making negative comments about their doctor. Secondly, expressing only positive views of their doctor may be used as a personal confirmation that the doctor was right. Negative thoughts about their doctor might jeopardize patients’ feelings of being reassured. Thirdly, since participating GPs were aware of this research project and the video recording and may have had affinity with its subject, GPs may have tried to enhance their reassuring actions, resulting in patients’ positive experiences. A positive outcome is that we have captured patients’ experiences of many different actions, yet we may have missed some of patients’ worrying cognitions.

Since we were not able to select from every practice consultations in which patients gave high ratings of their level of concern, we included several patients who indicated that they were only slightly worried. During the interview, however, these patients spontaneously mentioned several worrying and reassuring cognitions. We therefore think that the data comprise descriptions of worrying and reassuring cognitions given by patients showing a high degree and patients showing a lower degree of concern. This may well be interpreted as a strength of this study given that the current literature focuses mainly on patients with high anxiety, such as patients with hypochondriasis [30,33] and MUS patients [16,31,32]. The variety of the consultations is limited with respect to the absence of consultations in which GPs diagnose or suspect serious disease. It is likely that patients have other worrying and reassuring cognitions in case GPs express their concerns about serious pathology.

Another strength is that we interviewed patients about a recent consultation with their GP. This meant that the interviews remained very close to what actually happened during the consultations while the videotaped consultations were an effective stimulus for patients’ reflections. This reduced the risk of unreliable and incomplete answers, though the risk of patients constructing answers using hindsight knowledge is always present.

### Implications for practice and research

A good understanding of patients’ concerns is crucial for successful reassurance. It is not only reassuring in itself, it also gives doctors a focus for their efforts to reassure patients. Our results show a variety of cognitions underlying patients’ concerns. Once physicians are aware of the worrying cognitions patients have, they may be able to recognize patient cues pointing towards these cognitions. This is especially important in light of the finding that patients often did not mention all their concerns during the consultation and that these concerns were not addressed, in particular concerns related to treatment. Doctors should therefore pay attention to cues and concerns throughout the consultation, not solely during the opening phase. Awareness of the existence of ‘hidden’ worrying cognitions is essential for doctors aiming to effectively reassure their patients.

Furthermore, the reassuring core cognitions that were identified can support doctors in applying reassuring strategies. By not only describing reassuring actions but also the core cognitions supported by these actions and the worrying core cognitions counteracted by them, we aim to offer doctors guidance for strategies to reassure patients in a goal-directed manner. We do not recommend to apply all reassurance strategies in every consultation but aim to help doctors select the most appropriate reassurance strategy based on a proper exploration and understanding of the patient’s concerns.

In this small scale, qualitative study we were only able to describe patterns on the level of core cognitions. Specific underlying worrying cognitions, however, may require specific types of reassurance. It would therefore be interesting to conduct further experimental, systematic studies of the relationship between specific worrying and reassuring cognitions, in order to obtain additional insights regarding effective situation-specific reassurance. Future studies could explore specifically how different sorts of concerns are expressed during the encounter and factors that influence this expression, such as the presence/absence of a trusting doctor-patient relationship. Less intensive methods of data collection such as questionnaires would also allow the investigation of associations between patient characteristics (e.g. anxiety level)/GP characteristics and worrying/reassuring cognitions.

Using recently videotaped consultations as a stimulus for stimulated recall interviews appears to be an effective method of gaining insight into patients’ experiences and thoughts about the interaction with their GP. This method may also be useful for studies of patients’ perspectives on other important communicative aspects of doctor-patient consultations, such as patients’ feeling of involvement in decision-making.

## Conclusion

Both patients’ concerns about disease and patients’ reassurance are based on a variety of cognitions. What patients experience as reassuring seems to depend on their specific worrying cognitions. Gaining a thorough understanding of these worrying cognitions and tailoring reassuring strategies to them should be an effective way of achieving reassurance.

## Competing interests

The authors declare that they have no competing interests.

## Authors’ contributions

EG was involved in the design of the study, in the collection, analysis and interpretation of the data and drafted the manuscript. WV was involved in the design of the study and in the analysis and interpretation of the data and critically revised the manuscript. AM contributed to the data collection and analysis and was involved in drafting the manuscript. FB was involved in the interpretation of the data and critically revised the manuscript. TvdW and CvdV contributed to the design of the study and critically revised the manuscript. All authors read and approved the final manuscript.

## Pre-publication history

The pre-publication history for this paper can be accessed here:

http://www.biomedcentral.com/1471-2296/15/73/prepub

## References

[B1] Barry CA Bradley CP Britten N Stevenson FA Barber N Patients’ unvoiced agendas in general practice consultations: qualitative study Br Med J (Clin Res Ed) 2000 320 1246 1250 10.1136/bmj.320.7244.1246 PMC27368 10797036

[B2] McKinley RK Middleton JF What do patients want from doctors? Content analysis of written patient agendas for the consultation Br J Gen Pract 1999 49 796 800 10885083 PMC1313530

[B3] van Bokhoven MA Pleunis-van Empel MC Koch H Grol RP Dinant GJ van der Weijden T Why do patients want to have their blood tested? A qualitative study of patient expectations in general practice BMC Fam Pract 2006 7 75 10.1186/1471-2296-7-75 17166263 PMC1769380

[B4] Buchsbaum DG Reassurance reconsidered Soc Sci Med 1986 23 423 427 10.1016/0277-9536(86)90084-5 3749984

[B5] Lucock MP Morley S White C Peake MD Responses of consecutive patients to reassurance after gastroscopy: results of self administered questionnaire survey Br Med J (Clin Res Ed) 1997 315 572 575 10.1136/bmj.315.7108.572 PMC2127413 9302953

[B6] Stark D Kiely M Smith A Morley S Selby P House A Reassurance and the anxious cancer patient Br J Cancer 2004 91 893 899 15292934 10.1038/sj.bjc.6602077 PMC2409992

[B7] Fassaert T van Dulmen S Schellevis F van der Jagt L Bensing J Raising positive expectations helps patients with minor ailments: a cross-sectional study BMC Fam Pract 2008 9 38 10.1186/1471-2296-9-38 18590520 PMC2459169

[B8] Fogarty LA Curbow BA Wingard JR McDonnell K Somerfield MR Can 40 seconds of compassion reduce patient anxiety? J Clin Oncol 1999 17 371 379 10458256 10.1200/JCO.1999.17.1.371

[B9] Kessel N Reassurance Lancet 1979 1 1128 1133 86846 10.1016/s0140-6736(79)91804-x

[B10] Liénard A Merckaert I Libert Y Delvaux N Marchal S Boniver J Etienne A-M Klastersky J Reynaert C Scalliet P Slachmuylder JL Razavi D Factors that influence cancer patients’ anxiety following a medical consultation: impact of a communication skills training programme for physicians Ann Oncol 2006 17 1450 1458 10.1093/annonc/mdl142 16801333

[B11] Warwick HM Salkovskis PM Reassurance Br Med J (Clin Res Ed) 1985 290 1028 10.1136/bmj.290.6474.1028 3921095 PMC1418321

[B12] Sapira D Reassurance therapy. What to say to symptomatic patients with benign diseases Ann Intern Med 1972 77 603 604 10.7326/0003-4819-77-4-603 4642742

[B13] McDonald IG Daly J Jelinek VM Panetta F Gutman JM Opening Pandora’s box: the unpredictability of reassurance by a normal test result Br Med J (Clin Res Ed) 1996 313 329 332 10.1136/bmj.313.7053.329 PMC2351740 8760739

[B14] Meechan GT Collins JP Moss-Morris RE Petrie KJ Who is not reassured following benign diagnosis of breast symptoms? Psychooncology 2005 14 239 246 10.1002/pon.841 15386770

[B15] Petrie KJ Müller JT Schirmbeck F Donkin L Broadbent E Ellis CJ Gamble G Rief W Effect of providing information about normal test results on patients’ reassurance: randomised controlled trial Br Med J (Clin Res Ed) 2007 334 352 10.1136/bmj.39093.464190.55 PMC1800996 17259186

[B16] Rief W Heitmüller AM Reisberg K Rüddel H Why reassurance fails in patients with unexplained symptoms–an experimental investigation of remembered probabilities PLoS Med 2006 3 e269 10.1371/journal.pmed.0030269 16866576 PMC1523375

[B17] Donovan JL Blake DR Qualitative study of interpretation of reassurance among patients attending rheumatology clinics: “just a touch of arthritis, doctor?” Br Med J (Clin Res Ed) 2000 320 541 544 10.1136/bmj.320.7234.541 PMC27296 10688559

[B18] McDonald IG Daly J On patient judgement Intern Med J 2001 31 184 187 10.1046/j.1445-5994.2001.00036.x 11478348

[B19] Pilowsky I Aspects of abnormal illness behavior Indian J Psychiatry 1993 35 3 145 150 21743625 PMC2978482

[B20] Cegala DJ Gade C Lenzmeier Broz S McClure L Physicians’ and patients’ perceptions of patients’ communication competence in a primary care medical interview Health Commun 2004 16 289 304 10.1207/S15327027HC1603_2 15265752

[B21] Kenny DA Veldhuijzen W Weijden TVD Leblanc A Lockyer J Légaré F Campbell C Interpersonal perception in the context of doctor-patient relationships: a dyadic analysis of doctor-patient communication Soc Sci Med 2010 70 763 768 10.1016/j.socscimed.2009.10.065 20005618

[B22] Lapane KL Dubé CE Schneider KL Quilliam BJ Misperceptions of patients vs providers regarding medication-related communication issues Am J Manag Care 2007 13 613 618 17988186

[B23] Diefenbach MA Leventhal H The common-sense model of illness representation: Theoretical and practical considerations J Soc Distress Homeless 1996 5 1 11 38 10.1007/BF02090456

[B24] Coia P Morley S Medical reassurance and patients’ responses J Psychosom Res 1998 45 377 386 9835230 10.1016/s0022-3999(98)00047-6

[B25] Hirani SP Newman SP Patients’ beliefs about their cardiovascular disease Heart 2005 91 1235 1239 10.1136/hrt.2003.025262 16103576 PMC1769080

[B26] Leventhal H Leventhal EA Contrada RJ Self-regulation, health, and behavior: a perceptual-cognitive approach Psychol Health 1998 13 717 733 10.1080/08870449808407425

[B27] Lyle J Stimulated recall: a report on its use in naturalistic research Br Educ Res J 2003 29 861 878 10.1080/0141192032000137349

[B28] Watling CJ Lingard L Grounded theory in medical education research: AMEE Guide No. 70 Med Teach 2012 34 850 861 10.3109/0142159X.2012.704439 22913519

[B29] Tracy SJ Qualitative quality: eight big-tent criteria for excellent qualitative research Qual Inq 2010 16 10 837 851 10.1177/1077800410383121

[B30] Starcević V Reassurance and treatment of hypochondriasis Gen Hosp Psychiatry 1991 13 122 127 10.1016/0163-8343(91)90023-P 2037242

[B31] Gask L Dowrick C Salmon P Peters S Morriss R Reattribution reconsidered: narrative review and reflections on an educational intervention for medically unexplained symptoms in primary care settings J Psychosom Res 2011 71 325 334 10.1016/j.jpsychores.2011.05.008 21999976

[B32] Goldberg D Gask L O’Dowd T The treatment of somatization: teaching techniques of reattribution J Psychosom Res 1989 33 689 695 10.1016/0022-3999(89)90084-6 2621672

[B33] Warwick HMC Cognitive therapy in the treatment of hypochondriasis Adv Psychiatr Treat 1998 4 285 291 10.1192/apt.4.5.285

[B34] Andén A Andersson S-O Rudebeck C-E Satisfaction is not all–patients’ perceptions of outcome of general practice consultations, a qualitative study BMC Fam Pract 2005 6 43 10.1186/1471-2296-6-43 16242048 PMC1276792

[B35] Mikesell L Medicinal relationships: caring conversation Med Educ 2013 47 5 443 452 10.1111/medu.12104 23574057

[B36] van Ravesteijn H van Dijk I Darmon D van de Laar F Lucassen P Hartman TO van Weel C Speckens A The reassuring value of diagnostic tests: a systematic review Patient Educ Couns 2012 86 3 8 10.1016/j.pec.2011.02.003 21382687

[B37] Veldhuijzen W Ram PM van der Weijden T Niemantsverdriet S van der Vleuten CPM Characteristics of communication guidelines that facilitate or impede guideline use: a focus group study BMC Fam Pract 2007 8 31 10.1186/1471-2296-8-31 17506878 PMC1885263

[B38] Floyd MR Lang F McCord RS Keener M Patients with worry: presentation of concerns and expectations for response Patient Educ Couns 2005 57 211 216 10.1016/j.pec.2004.06.002 15911195

